# Control of foot-and-mouth disease in a closed society: A case study of Soviet Estonia

**DOI:** 10.3389/fvets.2022.828583

**Published:** 2022-08-01

**Authors:** Marko Kass, Arvo Viltrop, Julia Prakofjewa, Renata Sõukand, Raivo Kalle

**Affiliations:** ^1^Institute of Veterinary Medicine and Animal Science, Estonian University of Life Sciences, Tartu, Estonia; ^2^Department of Environmental Sciences, Informatics and Statistics, Università Ca' Foscari Venezia, Venezia, Italy; ^3^University of Gastronomic Sciences, Pollenzo, Italy

**Keywords:** foot-and-mouth disease, collective farms, veterinary treatment of cattle, distortion of the truth, Soviet Union veterinary system

## Abstract

Foot-and-mouth disease (FMD) is a dangerous infectious disease of even-toed ungulates, however since 1991, the European Union has banned preventive vaccination. During the occupation of the USSR, there were two outbreaks in Estonia: the first started in 1952 (at which time the barns typically housed about 20 cows); and the second began in 1982 (a period when barns typically housed several 100 animals). Neither outbreak was reported to the international community. At that time, it was also forbidden to talk about the disease in the internal media, and speakers could be punished. This study sought to find answers as to how the disease was treated and eliminated in the Estonian SSR, how infected animals and milk were handled, and if some of the methods used can be applied today. Written archival sources and 29 interviews with specialists remembering the outbreaks were used. Preventive slaughter of animals in the USSR was prohibited during the outbreak. As a preventive measure vaccination was used, traveling out of their counties by people were restricted and disinfection mats were used on the roads. In sick animals, udder wounds were treated with various wound ointments, such as zinc ointment, but also ointment made from boiled spruce resin. Birch tar was also recommended in the literature for leg treatments. Mouth wounds were washed with potassium permanganate solution. Workers used rubber gloves when handling sick animals. The barns were disinfected with lime and ash water. The milk from the diseased cows was pasteurized and given to calves, pigs, or diseased animals. Animals that did not recover were transferred to a meat processing plant. The meat was kept in potassium permanganate solution before processing and canned or made into sausages. When the disease was discovered, farm workers were locked in barns and released only when the disease had been eliminated. Such inhumane treatment could only be practiced in a totalitarian society.

## Introduction

Foot-and-mouth disease (FMD) is today considered to be one of the most problematic animal diseases, because it is very harmful to even-toed ungulate livestock farming. However, FMD has been endemic in Europe for a very long time, as early as the seventeenth century. The disease has become more common since the beginning of the twentieth century because of the widespread emergence of cattle breeding and increased trade in farm animals between regions (1). The biggest outbreak of FMD hit Europe in 1938–1939. The disease was detected at that time in all European countries.

The only country that was not affected by the disease at that time was the Republic of Estonia. This was achieved by imposing strict national preventive measures including import restrictions of animals from infected countries, disinfection of imported goods and hygiene requirements on migrant workers at border crossing points (2, 3). In 1939, the state railway company invested in the new washing unit for wagons, as livestock was transported by railway (4). The Veterinary Service in Estonia was aware of the preparation and use of foot-and-mouth disease vaccine used in Denmark and Sweden in the late 1930's (5). The first case of FMD was discovered in Estonia during World War II (6).

In early 1950's FMD was widespread in the Soviet Union (USSR), including in Estonia. Vaccination campaigns were conducted in parts of the country where the disease was spreading. In Estonia the disease was spreading mainly in the central and southern parts during these years (7). In the summer of 1956, a limited outbreak in smallholdings close to Tallinn was detected and successfully eradicated by killing susceptible animals in neighboring villages. This killing strategy was used for the first time here in the whole of the Soviet Union. Inspectors from Moscow were sent to check the local authorities and the farms to make sure it was implemented (8). In the 1960's, when there was a major FMD epidemic in other parts of the USSR, Estonia remained unaffected (9). The next time FMD was discovered in Estonia was 1982, when the epidemic that started in East-Germany reached Belarus and the Baltic Republics of the Soviet Union (10).

Sørensen et al. (11) proposed that in 1982 there was long-distance transmission of airborne virus FMD over the sea between Denmark and the former East-German, and that it was more likely transmission took place over water than over land due to the reduced surface turbulence over water (11). To prevent the disease, the first vaccinations were introduced in Europe as early as the 1920's (12). The European Union (EU) ceased preventive vaccination in 1991 after successful eradication of the disease in the member states. However, there is an ongoing debate if the vaccination should be more widely used, due to the animal welfare, economical, ethical and environmental consequences accompanying the killing strategy of FMD control (13, 14). Nevertheless, instead of vaccination, the main effort in disease prevention in the EU is based on bio-security measures and adapting animal husbandry to minimize the risk of spreading of infectious diseases (15, 16). Informing general public, involving people and the rapid availability of adequate information are also important during communicable disease outbreaks. In the case of a disease outbreak, it is very important to know where and how the infection was introduced to the farm. Thus, openness and transparency are very important in disease control (17).

Since the formation of the USSR in the 1920's, it began to shut itself down and create a “parallel society.” One example of concealing the real situation was a secret order from 1976 which described a “list of information prohibited from publication in the open press, radio and television broadcasts” (18). Among other forbidden topics was: massive diseases of farm animals with botulism, brucellosis, anthrax, plague, and foot and mouth disease. Thus, the correct data on the actual extent of infectious diseases were not published domestically nor was information provided abroad (19). Compared to a market economy, the USSR was built on a planned economy and the top-down plans were strictly enforced. Both meat and milk production had to follow a state-set plan. Because, in case of FMD, animal performance is reduced, this also put pressure on the food industry to make use of sick animals' meat and milk. Dairy cattle farming has been historically one of the most successful sectors of agriculture in Estonia for more than a century (20). In the wake of FMD disease in cattle that hit Europe after World War II, comprehensive texts on the outbreaks can be found only from the Western Europe, such as the report on the 1982 outbreak in Denmark (21). Nevertheless, there are quite a few reviews and analyses of the outbreaks in the USSR in the scientific literature. At that time, only general teaching about the disease and its control was conducted in the Estonia SSR (22, 23), but the actual extent of the disease was not reflected in the media. It has also not been studied if ethnoveterinary medicine was practiced in cattle farms in the Estonian SSR. It has been studied from the 1940's and for recent decades (24) but not in the soviet era.

As there are limited studies (7, 25) covering control of enzootic cattle diseases in Soviet Estonia, this study takes the deeper look at the spread of FMD and its control in a closed political system. In this paper, authors have tried to answer the following questions: (a) how and by what means the control of FMD in cattle took place in a closed society; (b) whether the methods by which the disease was brought under control are still relevant today; (c) whether, and to what extent, public information was provided and whether this lack of information led to alternative methods of treatment of FMD; (d) how the meat and milk of FMD affected cattle were used in the situation of general food shortages in the USSR.

## Materials and methods

### Description of the study area

**Figure 1:**
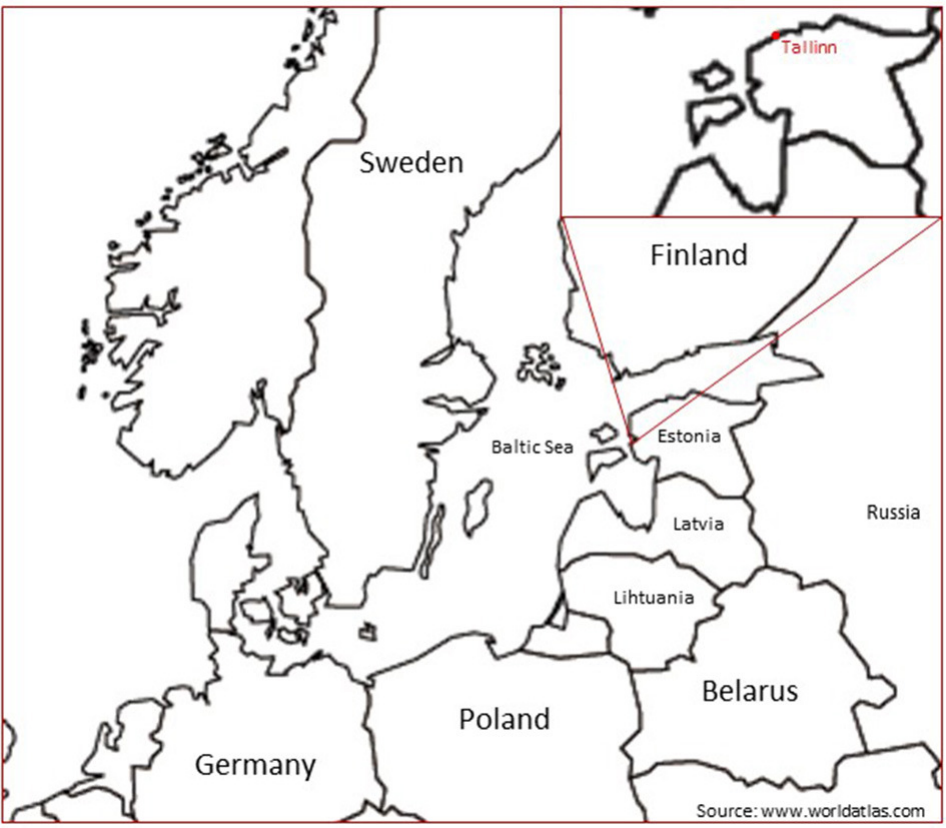
The map of Baltic Sea countries and Belarus.

Estonia is a country by the Baltic Sea (Figure 1). It borders Russia to the east and Latvia to the south. In 1710–1917, Estonia was part of the Russian Empire. From 1918 to 1940 the Republic of Estonia was independent (for a short period, in 1941–1944 it was under German occupation), and from 1940 to 1991 Estonia was annexed by the USSR. A public democracy movement peaked with the Singing Revolution in 1988, and in 1991 Estonia regained its independence (26).

**Figure 2:**
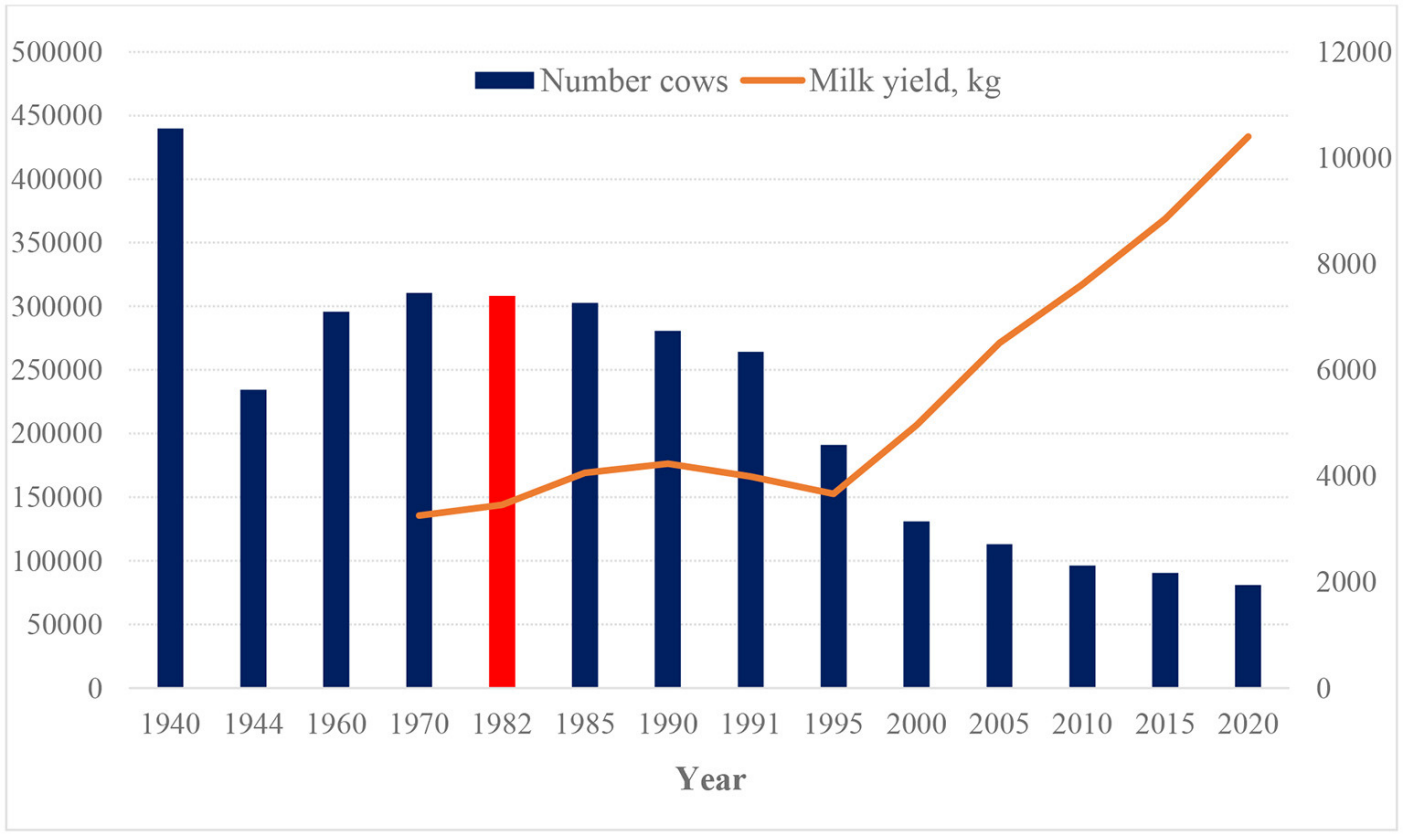
Number of cows and mean milk yield per cow between 1940 and 2020. There are no official data about milk production available from 1944 to 1960. Data from: www.epj.ee.

Estonia is located in a geographical area suitable for milk production and this became an important industry in the twentieth century. The number of cattle and milk production increased from the end of the Second World War (Figure 1). Considering the conditions at the time, reliable milk production statistics for the 1940's and 1950's do not exist. It was quite common that the work of inspectors revealed shortcomings in both livestock and dairy production records. For example, in May 1952, it was established that the reporting of livestock in the collective farms of the Estonian SSR was true in 74% of the collective farms for cattle, 80% for pigs and 91% for sheep. In addition, there were collective farms that did not keep records of the animals at all (27). Until 1940, considerable amounts of meat and dairy products were exported to Western Europe, but later these were sent to the USSR's domestic market (28). With the collapse of the USSR, this market also disappeared and the number of cows decreased significantly, although milk production per cow increased (Figure 2).

As mentioned above, Estonia was the only country in Europe that did not have FMD in 1938/39. Later, two outbreaks of FMD spread in Estonia: in the summer of 1952 (which lasted for several years) and in the autumn of 1982 and winter of 1983, although the Estonian SSR had been officially declared free of FMD since 1963 (29). In this connection, preventive vaccination against FMD was discontinued in the Baltic States in the early 1970's (30).

At the time of the 1952 outbreak, most herds were small, up to 20 animals. However, by 1982, herds of several 100 had already emerged. Thus, in 1952 the disease incidences were relatively small, but during the 1982 outbreak, the disease spread mainly only in Southern and Central Estonia, but it affected a higher number of animals. According to Peterson (7) there were 29,081 cattle infected by FMD during outbreaks in 1951–1953.

#### Censorship

In order to understand the general atmosphere in the USSR in the early 1980's, a few aspects should be emphasized. The period from 1964 to 1987 was called a period of stagnation in the Soviet Union, which started with the coming to power of Leonid Brezhnev in 1964 and ended with the XXVII Congress of the Communist Party in February 1986. That period could be defined as an unprofitable economy managed by the administrative-command system, a sloppy attitude toward state property and a lack of motivation of employees. Aggressive russification in the form of language policy, mass immigration of migrant factory workers to the republics (such as the Baltic States) and extensive censorship. The last meant that the Communist Party controlled all sorts of media including television, radio broadcasting, and newspaper and books. Soviet censorship combined pre- and post-censorship into one, resulting in ubiquitous, all-seeing and controlling permanent censorship (31). The media was selective in terms of content of good and bad news, meaning that the content of the newspapers should present the Soviet citizen in a good way, highlighting his/her achievements to support the goals of the party. News about accidents, disasters, criminal behaviors and crop failures in agriculture in the USSR, had to be ignored or covered only briefly and superficially in the media. It should also be borne in mind that the KGB (Committee for State Security; in Russian *Komitet Gosudarstvennoy Bezopasnosti*) intercepted absolutely all telephones (32), including those of veterinary officials. Therefore, the security service had probably an overview of FMD in this way.

In addition to the media, tacit censorship also applied to professional communication. This is another example of how censorship had to be comprehensive in Soviet society. In a situation where the authorities sought to nurture the Soviet people with common loyal behavior and worldview, in addition to the abundance of pro-regime propaganda and the ideologization of almost every sphere of life, including agriculture, an attempt was made to establish total control over all self-expression (33).

### Data collection

Information on the control and spread of FMD in Estonia during the Soviet era was collected from the electronic databases of libraries (e.g., https://www.etera.ee/, https://dea.digar.ee/), from the National Archives (https://ais.ra.ee/), the National Broadcasting Archive (https://arhiiv.err.ee/) and a database of Estonian language articles (https://artiklid.elnet.ee/). Electronic searches of newspaper, magazine websites, research databases (e.g., https://scholar.google.com/, https://books.google.com/, https://www.biodiversitylibrary.org/) were also conducted using keywords related to FMD and the Soviet Union. There is a lack of information about FMD outbreaks in neighboring soviet republics or in other parts of the USSR during the studied period. In those few cases the papers are either published by foreign researchers (34) or published in Russian (35) limiting wider conclusions.

The prevention and action of during the livestock disease outbreaks in USSR was established by the Veterinary Act, which was amended over the years. According to the veterinary legislation of the USSR, in 1968, the FMD was mentioned in the list of contagious diseases, in the occurrence of which a threatened zone—quarantine around the sick object (territory) was established. Also, following the developed instructions, animals affected by the foot-and-mouth disease were to be destroyed (without using meat, skins and other slaughter waste) (36). In 1965 (changed in 1971), the instruction on measures to prevent and eradicate the FMD was adopted. This document described the protocol for the essential measures to eradicate FMD in a diseased area, measures in an epizootic area, as well as measures to control FMD during the transportation of animals. The instructions also described measures for removing quarantine and subsequent temporary restrictions. The document also draws attention to efforts to protect free farms from the introduction of FMD. In 1968, guideline on how to collect, preserve, and ship FMD virus to determine its type was developed. In 1969 (changed in 1970), a temporary guideline for using a special foot-and-mouth disease vaccine was developed (36).

In 1974, the current Soviet veterinary legislation was amended to require dairies to clean and pasteurize milk from FMD-threatened farms. Finally, in 1974, the Chief Veterinary Administration of the USSR Ministry of Agriculture adopted regulations on vaccination to prevent foot-and-mouth disease (37).

The digital archive of the Soviet central newspaper Pravda (https://www.eastview.com/resources/gpa/pravda/) was searched in order to find public information. The analysis focused on one of the most influential of the print media—the broadsheet newspaper “Pravda” (in English *truth*), the official newspaper of the Communist Party of the Soviet Union from 1918 to 1991. This newspaper was chosen because of its relatively wide circulation (11 million copies) and the regularity and duration of its publications in the Russian language. Selected narratives exploited by Pravda in portraying some aspects of FMD were traced. Rough content analysis was performed on the newspaper, searching for texts dealing with the chosen theme. A total of 34 articles were selected. The small number of articles from one of the influential newspapers is clear. Nevertheless, we consider this a result in itself, an illustration of how little attention the Soviet media paid to FMD disease issues.

As there were very few written and archival sources, and the written sources of the USSR were often used in order to distort the truth, oral history was added as an important source. People who worked in kolkhozs and sovkhozs were interviewed (Supplementary material). The sovkhozs were under the state control, while the kolkhozs were a collective farm owned by the local people. In total, in February-April 2021, 26 people (17 males and nine females) were interviewed. The oldest respondent was born in 1928 and the youngest in 1964, mean age of male respondents was 71.2 and for females 70.9 years. The largest interviewed group based on (higher) education were veterinarians (*n* = 15) and zootechnicians/stockpersons (*n* = 9). One person was an agronomist and one was a biologist. Eight respondents were working as practicing veterinarians, and there was one farm manager, and one as stockperson at the farm. Six were university teachers (veterinary medicine or animal science), three researchers, three civil servants (state veterinary service), three (veterinary medicine) students, and one as zoo keeper during the outbreak of FMD in 1982. Most of the interviewees were retired or were working part time. The surveys were conducted in a semi-structured manner, based on a survey design. As the disease spread in Southern Estonia in 1982, most of the interviewers came from this region. In addition, four respondents had memories about the outbreak of the disease in the years 1952–1953. Respondents were informed that their data would be used for a scientific publication and that the data would be presented anonymously.

## Results

### General narrative of FMD in the Soviet Union: based on a survey of the newspaper “Pravda”

The first mention of FMD in Pravda was in 1924. Correspondents reported that FMD had been detected in the suburbs of Leningrad (currently Saint Petersburg). This led to the issue of a decree on the control against FMD, and preventive sanitary supervision over milk brought to Leningrad was strengthened (38). Furthermore, in 1925, FMD was involved in an international incident between Finland and USSR (39). In response to outbreaks of the disease in the Soviet Union, Finland closed the border to the movement of grain from the USSR to prevent the spread of FMD, thus stopping the export of bread. The article noted that the Soviets expressed indignation and checked the present state in the areas bordering with Finland. As a result, they put forward indisputable evidence of the absence of an FMD epidemic on the territory of USSR. They also emphasized the high level of development of veterinary medicine and guarantees for the non-spread of the disease. At the same time, continuing to deny the FMD epidemic on the territory of the Soviet Union, in 1926, Pravda urged the owners of sick animals to report the disease to the nearest doctor (veterinarian) immediately and not to consume raw milk or dairy products from diseased animals (40).

In the 1930's, the rhetoric of FMD narratives changed slightly. Correspondents noted specific outbreaks of the disease in different parts of USSR. Nevertheless, they assured their readers the disease passes quickly and without mortality (41, 42). But at the end of the 1930's and the beginning of the 1940's, the focus of attention of Pravda shifted to the countries of Western Europe. FMD was mentioned exclusively in world news chronicles (43–57). In the narratives, FMD was described as “a rampant disease that afflicts Western capitalist countries” with enormous negative consequences for their economies (44).

In the post-WWII period, Soviet correspondents referred to FMD as a bacteriological weapon used in the fight against USSR (58). FMD continued to feature only in the news section of the international press as an example of a disease spreading beyond the borders of the Soviet Union (59–67).

As for the territory of the Soviet Union, FMD was mentioned in the context of mandatory vaccination against the disease to prevent outbreaks and enormous economic damage (68). Also, Soviet correspondents noted the growth of international veterinary cooperation in the 1980's. So, for example, in 1983, within the framework of the Soviet-Afghan agreements, thanks to Soviet assistance in the creation of veterinary clinics and laboratories, Afghanistan was able to practically eliminate FMD on its territory (69).

In general, FMD was mostly mentioned in the pages of Pravda in the context of international news. No mention was found of FMD in the peak years of its spread in the Soviet Republic of Estonia.

### Reflection of the disease that began in 1952 in the Estonian media literature and interviews

The first report of the disease did not appear in a national newspaper until July 1st 1952 (70). This was published by the young veterinarian Heino Mikk. He was one of the best-known speakers, and he published several articles later on this topic (71–73). It was forbidden to write in newspapers that the disease was spreading in Estonia, only how to recognize the disease and what control methods must be applied. It remained the only nationwide public announcement. Recommendations were also shared later in dozens of agricultural publications, so-called propaganda newspapers (74–76). Although thousands of copies of these newspapers were published, they had virtually no readership at all, people were not interested in empty, large-typed propaganda, and therefore these newspapers were not bought or subscribed to (77).

The first documentary in which the real extent of the disease that struck Estonia in 1952 was discussed, was not broadcast on television until March 7th, 1988 (77). This was made possible by the innovation (*perestroika*) and disclosure (*glasnost*) reforms initiated by Mikhail Gorbachev (became the leader of the USSR in 1985). In the TV programme, veterinarian Heino Mikk (1924–2001) talked about his experiences in controlling the disease in 1952. He had just graduated from university as a veterinarian and had little experience at that time. He later became the chief veterinarian of the entire Estonian SSR in the field of disease control. He reported that the first outbreaks of the disease appeared in Estonia in the winter (January-February) of 1952. There were very few veterinarians at that time and they had no knowledge of the disease or its control. Although Mikk claimed that veterinarians did not have previously experienced with the disease, it can be assumed that they could have studied it at university. It was also forbidden for people involved in disease control to talk about it in the media. These were possibly the reasons why the disease began to spread widely; the damage caused by the disease at that time was colossal. The measures initially proposed were disproportionately stringent: for example, all movement in rural areas was banned, even on major roads. Farm personnel had to stay in the barns for a month, all gatherings and entertainment events were prohibited for more than half a year. However, the killing of infected animals was considered unthinkable.

Jaan Roos (1888–1965), an internally displaced person (protester against the Soviet regime), also described the real situation at that time in his diary (78). His diary of these years (1951–1952) was published only in 2004. By March 1952, the FMD situation in some regions of Southern Estonia had become very serious. In the quarantine area, the roads were closed and guarded, and any movement inside the area was punishable with 2 years in prison. However, people still moved in some places along secret paths during the quarantine. Even in areas where there was no disease, collective farms workers were forbidden to communicate with strangers. There were rumors that the disease had been brought to Estonia during the winter from Lithuania with sugar beet (78). The disease was reported to have been contracted by 14 people (77, 79).

Mikk and Endel Aaver (1927–2009), a senior researcher (virologist) at the Estonian Institute of Animal Husbandry and Veterinary Research, and a former adviser to the Ministry of Agriculture, Ants Laansalu (1938–2011) recalled that the disease which began in 1952 was very serious (77). However, more animals died from massive feed shortages than from the disease. In 1952, the number of cows decreased by 6%, of which about half died directly from the disease. There was practically no feed for cattle in the kolkhozes during the spring-winter period. Weakened cows were lifted up by rope every morning. Some animals stayed on the ropes for the whole day because they could not stand on their own.

Only four persons interviewed had personal memories of the outbreak that occurred in the early 1950's. Two of them had been involved as veterinarians in the 1950's. Now 82 years old a zootechnician (who worked in the 1950's) recalled that people wondered at the time how the disease suddenly came to Estonia and why. There had never been such a disease in Estonia before according their knowledge. Since most of the respondents had directly or indirectly experienced the outbreak, in general, their recollections were similar in several respects, such as the procedure for quarantine, farm lockdowns, disinfection mats on roads, militia guards at farm gates and so on. A zootechnician (aged 82) recalled the control of the disease as follows: “When I studied to be a zootechnic at vocational school in the 1950's, all the students were taken to the dairy farms. There we had to wash the mouths of the animals with a solution of potassium permanganate. The animals were kept for as little time as possible in the barns during the summer. The reason was because the cows' hooves become more damaged inside in the wet manure. The barns were disinfected with lime and ash water. Ash was gathered from the people by the village.”

### Estonian media coverage of the 1982/1983 disease and written sources

There are no reports or news stories in the newspapers about the FMD outbreak in 1982/1983. The first announcement about the FMD appeared in a national newspaper in November (80) and December (81). Again, it was not written that there was any disease in Estonia at all, but information was given as to how to recognize and control the disease. People were warned that the disease could also be transmitted to humans, yet it was said that processed meat (canned and sausages) from sick animals could be eaten and pasteurized milk could be drunk.

Due to the FMD epidemic in England in 2001, the topic of FMD became very popular in the media. In 2001, detailed recollections of the 1982 outbreak also appeared in the press. In one of them, a worker of the Vändra Kolkhoz recalled his experiences and comments were taken by Endel Aaver and an interview with Chief Inspector Mati Loit at the Veterinary Government of the Estonian SSR (79, 82). The topic was also covered a few years later where the manager of the cattle unit of the Laatre Sovkhoz (83) recalled his experiences. The harsh control techniques in the UK were similar to those in Estonia in 1982. People were locked in barns such in Laatre dairy farm (South Estonia), nearly 30 workers were enclosed for 42 days. Also, in Vändra cattle farm 10 workers were isolated for more than 30 days. Employees were not told they had to stay in the barn for weeks so at first, they did not take their personal belongings with them. The gate doors of the cattle farms were guarded by militia 24-h a day. There were also strict rules inside the farm, men and women slept in separate rooms and drinking alcohol was forbidden. Also, vaccination of the animals started immediately. The milk from diseased cows was first added to dung for disposal, later it was pasteurized on site and fed to calves and vaccinated cows (in some cases to pigs). Hundreds of sick animals were moved to a meat processing plant after the end of the quarantine period. The abattoir was then subsequently disinfected. Everything had to be kept secret. For example, a veterinarian who accidentally mentioned the disease on the radio was punished (79, 82, 83). Rumors spread that it was an experiment and that the animals were intentionally infected. The reasons for the rumors were: the disease spread among the most advanced and hygienic farms (disinfection mats at the farm entrance, warning signs at the gates), previous secret searches on the farm, unknown people with large sums of money. The manager of the cattle unit of the Laatre Sovkhoz remembered that the situation on the disease had to be reported every day to unknown anonymous Russian-speaking officials. Disease control specialists were also brought to Estonia from other parts of USSR (83).

Aaver and Mati Loit said that in 1982 a total of eight outbreaks occurred in Estonia. According to Aaver, the FMD arrived in Estonia from Belarus through Lithuania and Latvia and reached the sea in the Western part of Estonia. However, there is not much information about the outbreaks in Lithuanian and Latvian SSR in 1982 (84), but in years 1986 and 1987 (85). In addition, Aaver pointed out that more than 30 people from Moscow, who had seen FMD in USSR almost every year, came to aid Estonian specialists at that time. Analysing the wind directions at that time, it has been concluded by researchers that the disease could be spread by the wind. It was found that when the direction of the prevailing wind changed, the infection also decreased (79, 82, 83).

#### Foreign media coverage of the disease that hit Estonia SSR

The 1982 outbreak reached the foreign media very quickly. During World War II, almost 80,000 Estonians fled abroad for fear of repression by the Soviet authorities. The largest diaspora was formed in Sweden. Estonians abroad maintained active communication with relatives who remained in their homeland. Thus, the first warnings of the disease in November in the national newspaper of the Estonian SSR reached numerous foreign Estonian newspapers in December 4: in Sweden (86), Canada (87), the USA (88), and Australia (89). However, in addition to official sources, foreign Estonian newspapers also relied on direct sources, which were letters from Estonia. Thus, the actual extent of the disease and the control measures were known abroad quite quickly and in detail. The disease spread from Russia to Lithuania and Latvia in the spring, and from there to Estonia in the autumn (90). The letters from Estonia SSR, also show that there was a great shortage of food in Estonia and ideological pressure was increasing (88). It is noted that, due to FMD, foodstuffs were no longer transported (exported) from Estonia SSR to the domestic market of the USSR and therefore more goods were available in the shops in Estonia (91). The few tourists that visited the Estonian SSR also reported on the control and spread of the disease (91). It was repeatedly emphasized that the Soviet Union denied any outbreak of the disease. However, Sweden suspended imports of reindeer, elk and bear meat from the USSR (92) and Finland suspended the ferry service (91). However, the foreign press did not know the whole truth at the beginning. It was initially thought that infected animals would be killed in Estonia and then burned or buried. However, this was not done.

As early as March 1983, a report appeared in US infectious disease reports mentioning an outbreak in the Baltic Soviet republics. However, there was no overview of the actual situation as reported. “However, detailed information is not forthcoming from this region” (84) but the June issue states that “The USSR has denied any outbreaks occurring in its Baltic regions / — /” (19).

#### Analysis of interviews on the 1982/1983 outbreak

##### The problem of secrecy and how the information was exchanged and rumors

Two respondents (a zootechnian aged 69 and a veterinarian aged 71) who worked at the country's first large farm in South Estonia highlighted that the dairy farm had been closed for 43 days, from November to the end of December, including Christmas. One of them believes that the disease was brought to the farm intentionally, in other words for training purposes. Respondents added that the disease was noticed when two cows began to exhibit the symptoms of the disease: bite their mouths, producing foam in mouth and intensive saliva flow.

One reason for intentional outbreak was that the farm was the production farm of the state animal science research institute, which had modern furnishings and living rooms for the employees (sauna, kitchen, rest rooms), which were not available on other collective farms. This played an important role in combatting FMD on the farm, and about 14–16 people were trapped in the barn. In addition to treating and feeding the animals, staff and clothing and other supplies had to be disinfected on a regular basis. The workload was several times higher than usual because the staff could not leave the farm. After the closure of the farm, beds and mattresses were brought to the staff. Food was provided on a daily basis, and one milking lady began to work as a chef. At that time there was a very strict order on the territory of the farm, there was no access for strangers either. There was a 24-h militia guard at the gate. Those trapped on the farm did not see their family members, children or other relatives. This was very difficult psychologically for all the staff in addition to the physical load.

Another reason of intentional outbreak given by respondents was that somebody called every day at 9 o'clock and asked in Russian about the number of animals that were sick and if there were any dead animals. This raised doubts as they were not told who it was who called every morning and asked for these details. It is important to highlight that it was very common in the Soviet Union to conduct training exercises to practice actions for major accidents, including outbreaks of animal diseases, as part of civil protection exercises. Although there are several indications in the respondents' answers that the animals were intentionally infected with FMD, it should not be forgotten that the disease had been detected in the neighboring republic a few months earlier. In the north of Latvia SSR; Tallinn Zoo was closed in 1982 for about 6 months. As the zoo was visited by a significant number of Latvian visitors each year, the zoo had to be closed to avoid FMD. One of the interviewees mentioned that Tallinn Zoo had one of the most representative collections of Bovines and mountain goats in the world. The 1982 FMD outbreak also meant for the zoo that animal feed (such as hay) had to be purchased from Western Estonia instead of the usual southern regions, as there was no imminent threat of FMD from the west.

##### The effect of the restrictions on daily life of the farm employees

Another major issue that emerges from the survey is the various restrictions, such as road closures and the restrictions on people in the farm and the resulting trauma. One of the respondents (agronomist, aged 76) “people had fear because everyone had animals in their household.” A former veterinarian (aged 84) reported: “People knew about the outbreak in southern Estonia. People were very afraid. The authorities also vaccinated one-cow farmers (the authors—cows were kept in virtually every household in rural areas).” A zootechnician (aged 74) narrated: “Cabbages and turnips remained in the field in the affected area because movement in that area was prohibited.” A former farm manager (aged 69), remembered: “14–16 people were locked in on the farm. There was a lot of work. There were militia at the gate. The children could only be seen through the fence, as many as the spouse could hold.” Several respondents also stated: “The restrictions were very strict at the time. In some places, roads were even dug up to distract traffic.” “At the border of the districts there were disinfection mats, the driver had to come out of the cab, and walk across the mats. The shoes smelled afterwards. There was constant guarding of those mats. Almost half a year, but it varied by region; in general, restrictions lasted almost half a year” (agronomist, aged 76). A veterinarian (aged 66) stated that “veterinary students were taught at the university model-based disease control: blocking of roads, bridges and farms during potential animal disease outbreaks. Control instructions were described in detail. Informative posters near farms were common. Barriers at farms and on roads too.”

It was also mentioned on a few occasions that the manure of the sick cows had to be stored separately, which could not be used as organic fertilizer for years. In some places, dead calves were also burned on bonfires near the cattle unit.

##### Treatment of sick animals with symptoms or preventive measures

When the udders had sores, the milkers protected their hands with rubber gloves while milking. Udder wounds were smeared with zinc ointment, but also udders smeared with ointments. Outside the kolkhoz, people were only allowed to have one cow at home, so the animals were kept in barns at home very carefully. During the period of FMD the milker was detained on the farm, and food was brought into the barn. Some cleaned the barns during the outbreak with juniper smoke and the Russian Orthodox believers near Lake Peipus also brought water consecrated from the church and watered the animals with this. Folk medicine was no longer used in the kolkhozes, but there are some indications of this being used by single cow owners. A former veterinary scientist (aged 74) said: “The cow was still being treated by a veterinarian, folk medicine was not used at the time.” FMD vaccine was thought to have been available in the early 1980's as the disease spread across the Soviet Union. This was confirmed by several respondents, one (zootechnician, aged 69) stated that: “Vaccination started immediately in cases of the disease. No animals were killed as a result.” This involved a great deal of effort. The cows that were caught were heavily blistered and could not eat normally and it was necessary to prepare a mixture of boiled milk (which could not be sold for human consumption) and grain meal, as it was not possible to feed hay because of the sharp stems irritating the blistered mouths of the cow. A zootechnician (aged 69): “It was painful for the cow to eat hay. They ate a little silage as it was softer.” This meant that sick animals had to be fed several times a day to avoid loss of body condition. A veterinarian (aged 71) added: “The cows' mouths were rinsed with a copper preparation. Blisters and sores were also present on the hooves, which were smeared with ointments.” It was also mentioned on several occasions that, because the animal was in pain, it led to thinness. It also meant a significant decline in milk yield, even after the cows had recovered. A zootechnician (aged 69) said: “The cows' milk yields did not recover after the end of the quarantine. However, the animals themselves recovered, their body weight returned to normal and the cows were alive.” Interviews revealed that one large farm in South Estonia was able to prevent deaths due to huge efforts by the farm personnel, but another farm in the same region had more serious cases, resulting even in dead animals.

One veterinarian described the activities during high risk of FMD at a dairy farm in Central Estonia. The story of one veterinarian (aged 85) was as follows: One day in year 1982, at 4 pm, the head of the Tartu District Veterinary Service convened the chief veterinarians of all the farms in Tartu district and distributed vaccines. By the evening of the same day at 11.30 pm, more than 3,000 cattle from our state farm, including 980 cows, had been vaccinated. Everything happened at lightning speed. This prevented the greatest danger. Tartu district is one of the few where there was no foot-and-mouth disease. In any case, the Chief Veterinary Officer of Tartu District later received a letter of honor and/or a letter of thanks from the Veterinary Government of the Ministry of Agriculture. The veterinarian added: “We were given information about foot-and-mouth disease at the monthly meetings of the chief veterinarians, and this was discussed at several meetings. As mentioned above, there were 3,000 cattle in the state farm, and about 12,000 piglets were produced each year. I had a total of 110 livestock workers, including three veterinarians and one assistant veterinarian. Stockpersons, together with the respective regional department heads, made every effort to prevent the spread of the disease. There were disinfection mats in front of both the large farm and each of the smaller barns, as well as a very large and long disinfection bath, where the long semi-trailers bringing concentrate feed could completely wet the wheel tires with disinfectant. These lorries were also disinfected by a trained specialist. A separate topic was the protective clothing of stockpersons and others, to which we paid more attention than before. The fact that many workers had cattle and pigs at home was alarming. The infection could potentially have started there as well. The FMD of 1982 passed our state farm, but the older milking ladies and workers remembered the beginning of the collective farm foot-and-mouth disease in 1952, when the children could not see their parents and the livestock workers had to live and sleep in the stable for weeks.”

##### Use of milk from sick animals or meat from recovered animals

A zootechnician (aged 69) mentioned that “many animals suffered but recovered, while milk yields declined remarkably.” The milk was not allowed to be transported out of the farm, it had to be fed either to youngstock or to cows. For this reason, a boiler was brought to the farm to pasteurize the milk. However, the affected animals sooner or later had to be culled. The interviewee added: A total of 92 animals were taken to the slaughterhouse, but in fact worse and thinner cows were sent there at first. However, a year after the disease outbreak, a large number of the animals had to be taken to a meat processing plant, a total of 1,400 animals. This was required by the veterinary regulations. The meat of the affected animals was made into canned food, i.e., the meat had to be heat-treated. And a stockperson (aged 82) remembered: “Animals who were weakened by the disease were transferred to a meat processing plant. The meat was kept there for a day in a bath with a solution of potassium permanganate before it was processed.”

The outbreak of the disease meant that foodstuffs of animal origin could no longer be placed on the domestic market of the USSR and more of these goods were sold in the shops of the Estonian SSR. For example, one respondent (researcher, aged 83) mentioned: “Foot-and-mouth disease closed the borders of Soviet Estonia. The butter came back for sale in food stores because it was no longer allowed to send parcels with food products to other Soviet republics. At that time, it was common that people of Russian speaking nationalities (e.g., workers at large factories) sent a monthly package of food products to their relatives to other republics.”

## Discussion

Even the most prestigious Soviet magazine “Socialist Agriculture” (in Estonian *Sotsialistlik Põllumajandus*) did not address the topic of FMD, only good news topics were written about the exceeding of 5-year plans, increasing production in kolkhozes, effective application of research results in practice etc. However, it is known that due to the well-established system of the veterinary service, the information flowed quickly and the veterinarians of the whole country were aware of the seriousness of the situation. However, as can be seen from the respondents' answers, the population was also aware of the situation, although FMD was not covered in the newspapers or on television (all controlled by the state). The severity of the situation was also signaled by roadblocks at the borders of the district, disinfection baths and even militia guards to make sure that all vehicles and passengers were disinfected to prevent the spread of the disease. However, as mentioned by the interviewees on several occasions, there was minimal public talk about the disease, the epidemics were strictly regulated and there was no need to speak on the subject. Disease control was strictly regulated by the state, with its own chain of command and action plans.

The first teachings on the control of FMD to the general public, especially farm workers, were published in 1952. Thus, at the end of 1952, veterinarian Šolom Špungen (1908–1964) published a 32-page brochure describing the disease and control techniques. It had a print run of 5,000 copies (23). He also edited an informative color poster (size of 58 × 87.5 cm) and had a circulation of 3,000 copies. It was freely distributed (93).

We also looked at whether there was any attempt to replace the shortage of synthetic medicament in the USSR. Alternative remedies were recommended to veterinarians in the 1980's. For example, veterinarian-pharmacologist Richard Lumi (1905–2000) advised veterinarians that for animals with FMD, the claw guards and the interstitial skin of the claws should be lubricated with birch tar (94, 95). At that time, birch tar was also widely recommended for the treatment of human skin diseases (96). Lumi also recommended disinfecting barns with Sodium hydroxide, formaldehyde solution or chlorinated lime solution during a disease outbreak (94, 95).

Those respondents who remembered the outbreak of FMD in the early 1950's in the Estonian SSR said that animals were generally suffered little with the virus. Techniques were even used in which healthy animals were infected with saliva from a diseased animal. Similar treatment has been practiced by Fulani herdsmen in Northern Nigeria, who sometimes move their cows upwind of infected animals to prevent the FMD from spreading, and sometimes they move them downwind to expose the cattle to the disease, knowing that a mild case of the FMD will not be fatal and will confer immunity (97).

It seems that results of collectivisation in the 1940's also meant that folk medicine began to disappear from local livestock production. For example, plants such as *Crataegus oxyacantha* L., *Inula helenium* L., which had been used to treat cow's foot diseases in previous times (24), had already been replaced by ointments from veterinary pharmacies by the early 1950's. However, scientific experiments with folk medicine have been successfully conducted in India and Kenya. The treatment of FMD wounds with honey ointment is particularly promising (98, 99). Herbal treatment of FMD in domestic animals is a common practice in Africa, where the disease is more prevalent today. In Kenya, for example, *Ricinus communis* L. leaf is chopped and mixed with water, then used for topical application (100); *Allium cepa* L. bulb are chopped and given with salt for 2 days, for ruminants by mouth (101); *Stephania abyssinica* (Quart.-Dill. and A.Rich.) Walp. whole part plant is pounded, water is added, then drenced (102).

Another aspect is the fact that the Soviet authorities used agriculture as part of the propaganda. In the years 1949–1950, the elimination of family farms and farmer unions took place, and rural people were forced to participate in the formation of collective farms. However, in the late 1940's and 1950's, agriculture was characterized by low productivity as the state farms were plagued by a chronic shortage of labor and agricultural machinery (103). As the Soviet authorities used all means and resources to legitimize their power and to prove the uniqueness of Soviet agriculture, agricultural exhibitions gained unprecedented ideological significance in Soviet society (104). Due to the widespread outbreak of foot-and-mouth disease and poor weather conditions in 1952 (7, 70), agricultural exhibitions were banned in half of the districts in republic, but there was not the slightest indication in the newspapers about any decline in production in collective farms (104). Another reason for the abandonment of agricultural exhibitions was the massive mortality of livestock and the foreseeable consequence of the spread of animal diseases in such conditions. Between 1948 and 1950, eight to nine piglets died in the state farms of the Estonian SSR. And it was not surprising that illegal deaths of cattle often occurred in this situation (103).

As the USSR dealt with control centrally during the FMD outbreak of 1982, the Estonian archives today do not contain any reports or reports on the actual situation since then. They may be in Russian archives. However, for the 1952 disease outbreak, the Estonian archives contain many regional reports. Why was it necessary to address the 1982 outbreak at the highest level? The USSR delegation reported at the FAO 25th Foot-and-Mouth Disease Session in Rome, Italy, on April 12–15, 1983 that:

*In the USSR, there have been rumors of FMD outbreaks in the Baltic States which have been thought to be free of FMD. The USSR Veterinary Authorities informed the Secretariat that an extensive prophylactic vaccination programme had been carried out in November 1982 and that no cases of FMD had occurred in this area. Five million cattle and four million sheep have been vaccinated with OAC vaccine in the Baltic provinces* [(105), p. 16].

The same report showed that in 1981 there were 14 outbreaks in the USSR and in 1982 there were nine outbreaks. They were said to be caused by 2-O, 1-O, 2-O1, and 1-O1 viruses (105). According to United States Department of Agriculture (19) report USSR denied any outbreaks occurring in its Baltic regions and cattle were vaccinated in fall 1982 to decrease the risk of infection. It is known that there were at least eight outbreaks in Estonia in 1982 alone, and before that the disease spread in Latvia and Lithuania (79). However, the veterinary officials from the central veterinary office of the Estonian SSR at a time of 1982/83 epidemic report in 1992 that this epidemic was caused by the serotype A22 (9). From the FAO report (105), it appears that this type of virus spread at that time only in Turkey. It is not clear what was the source of information for the Estonian veterinary officials regarding the type of the virus spreading in Estonia during the 1982/83 epidemic. In the FAO report (105), the representatives of USSR claimed that animals were vaccinated with a trivalent vaccine in which A22 was only one component. The others were O and C. Only O was needed for protection. Given the secrecy of the time, Estonian officials might have not known the virus type that was actually circulating in Estonia. Maybe they were able to draw this conclusion from the label on the vaccine bottle, where A22 was listed first. The second possibility is indeed that the virus that spread from East Germany (serotype O) never reached the Baltic republics and the epidemic was caused by a strain circulating in USSR without being reported to international organizations (A22).

As early as in the beginning of 1960's, USSR researchers under the Ministry of Agriculture were instructed to develop bio-weapons against livestock and crops. These bioweapons with pathogens had to be able to be attached to the bombers and be sprayed over large areas. One potential bioweapon virus was FMD (106). FMD has been considered the ideal source of bioweapons, also outside the USSR. It is thought that a number of outbreaks in Asia, as well as in England in 2001, may have arisen from the use of biological weapons (107). It cannot be ruled out that a possible leak of the virus from the laboratory was concealed. There have been several cases of laboratory leakage in the European Union (108). It can be assumed that such things happened in the USSR as well. In addition, it has been assumed that the development of biological weapons related to livestock production continued after the collapse of the USSR (109).

Respondents stated that such thorough closures, with staff locked in at the farms, i.e., restrictions on human freedoms, could only be implemented in a totalitarian regime. As one respondent (veterinarian, 81 years old) put it: “Only in a totalitarian state can animal diseases be effectively controlled.” In addition, it is clear from the respondents' statements that “in 1952 restrictions were wider, everything was locked. There were militia guards at the cattle farms.” The situation was often very critical, as “Already in the 1950's, people at risk of death were kept away from stables and children could not see their mothers for weeks.”

One important aspect to point out in the example of the Estonian SSR is that the meat of the affected animals was used for human consumption as there was already a great shortage of the most common food products at that time, there was no question of burying or burning cows infected with FMD. Besides, in May 1982 the Central Committee plenum of the Communist Party set a goal for the new Food Programme which was to promote and improve the productivity and output of agriculture in the Soviet Union.

By the early 1980's folk medicine was no longer practiced on cattle, at least not in the collective and state farms in Soviet Estonia, as was described by several respondents. There were one or more veterinarians working in larger dairy farms, and there was a chief veterinarian in each Kolkhoz. There were creams or solutions prescribed by a veterinarian which were used to treat blistering and hoof problems in dairy cattle in cases of FMD.

There is no clear evidence if there the vaccines before the 1982 outbreak. One of the respondents mentioned that, when the FMD outbreak took place, the vaccines were delivered by the authorities from Moscow. Due to the good functioning of the veterinary service at the time, and the very strict chain of command, the vaccine reached the regions fairly quickly.

As the outbreak of the disease in Estonia in 1950 showed, when the herds were small, the impact of the disease was smaller. The most important factor was economic instead: the transition from a free market economy to a planned economy and the resulting difficulties. Before World War II, Estonia had a market economy and it was only during the occupation by the USSR that the planned economy came into being. Our study found that newly opened large farms performed relatively well in controlling FMD, largely due to existing infrastructure. However, we do not have information on how farms with fewer cattle (e.g., <100 individuals) would have been able to control the disease. As D'souza and Ikerd (110) acknowledge, smaller farms could be more sustainable. To change the system, it is necessary to change the narratives [see also (111)]. One of the narratives that needs to be changed is that the milk of sick animals is not suitable for drinking and meat is not to be eaten. As the experience of the USSR has shown, it is possible to produce both meat and milk sustainably during an outbreak. The solution would be smaller slaughterhouses and smaller dairies. Smaller ones are more flexible and can also adapt better to special conditions.

## Conclusion

The paper describes the events of 40 years ago in the Estonian SSR, dealing with the outbreak of foot-and-mouth disease that began in southern Estonia. The focus was coverage of the outbreak of FMD in the autumn of 1982 in the local media. As agriculture was a priority for the entire Soviet Union, the Communist Party's narrative was to treat agriculture only for good. The latter meant that the party watched very closely how agriculture was presented in the public media.

Due to the growth in demand for animal origin products in the Soviet Union, dairy farming in the Estonian SSR developed significantly in the 1970's and 1980's. In the first half of the 1970's, the first large cattle barns with a capacity of up to 1,000 animals were built, which also included the corresponding infrastructure for personnel. When interviewing farm managers and animal husbandry specialists at the time, we tried to get an adequate overview of what happened in the autumn 1982. Unfortunately, this paper failed to explain why FMD broke out on large farms. However, some of the interviewees hypothesized that dairy farms in the Estonian SSR, as the most advanced in the entire Soviet Union, were used as a testing ground to monitor the development of the disease and its control mechanisms. The main argument to support this claim was that large dairy farms had the appropriate infrastructure to allow staff to remain on the farm during the quarantine period.

Based on the newspapers and scientific publications of that time, it might be assumed that there was no FMD in the Estonian SSR in 1982. However, most of the republic knew that the traffic restrictions on the roads were related to the disease outbreak in South Estonia. At the same time, the outbreak was covered in the media of neighboring countries, which described them in some detail. Thus, the present study is another example of the narrative of the system in the Soviets, where troubles and problems had to be silenced and only progress and victories could be publicly affirmed, which the Communist Party largely wrote at its own expense.

Methods used in a totalitarian system cannot be used in a free society. The trauma that people were forced into the barns (more than a month in some cases) also affected the next generation. Preventive actions are the most important in the control of FMD. It appears from the interviews that at that time people were generally more accustomed to the restrictions of the state, because that was the (Soviet) time.

## Data availability statement

The raw data supporting the conclusions of this article will be made available by the authors, without undue reservation.

## Author contributions

MK, AV, and RK conceived the study. MK, AV, JP, and RK gathered the data. RS supervised the study. MK and RK analyzed the data with the help of the co-authors. MK drafted the first version of the manuscript which was then reviewed, edited, and approved by all authors. All authors contributed to the article and approved the submitted version.

## Funding

The postdoctoral research fellowship of MK at the Scotland's Rural College was supported by the Estonian Research Council (PUTJD968). Research by RK, JP, and RS was supported by the European Research Council (ERC) under the European Union's Horizon 2020 research and innovation programme (Starting Grant project Ethnobotany of divided generations in the context of centralization, Grant agreement No. 714874).

## Conflict of interest

The authors declare that the research was conducted in the absence of any commercial or financial relationships that could be construed as a potential conflict of interest.

## Publisher's note

All claims expressed in this article are solely those of the authors and do not necessarily represent those of their affiliated organizations, or those of the publisher, the editors and the reviewers. Any product that may be evaluated in this article, or claim that may be made by its manufacturer, is not guaranteed or endorsed by the publisher.
