# Supplementary material for: Control of foot-and-mouth disease in a closed society: A case study of Soviet Estonia
Source: Front Vet Sci. 2022 Aug 1;9:828583. doi: 10.3389/fvets.2022.828583 (PMC9429994; doi:10.3389/fvets.2022.828583)
Supplement: Supplementary file 1 [file Data_Sheet_1.pdf]

Supplementary

SURVEY

Open questions asked during interviews

1. Which outbreaks of infectious bovine diseases do you remember in Soviet Estonia from 1940-1990? (a) foot-and-mouth disease in 1950s; (b) foot-and-mouth disease in the 1980s; (c) other (please specify).

2. How did you find out about the diseases (outbreaks)? (a) acquaintances; (b) a newspaper; (c) by radio and television (foreign media); (d) veterinarians (colleagues); e) other (please specify?).

3. Did you had a contact with cattle (other farm animals) during the FMD outbreak? (a) we had livestock in the household; (b) was a worker at the farm (e.g. a zootechnician); (c) was a practicing veterinarian; d) was both a practicing veterinarian and had livestock in own household e) was working in the veterinary service f) other (please specify).

4. Do you remember what kind of restrictions were set? (several options) a) road closures; (b) restraint; (c) disinfection of the body during movement from one division to another; (d) an obligation to remain silent about the outbreak; (c) other (please specify).

5. Do you remember the so-called conspiracy theories that spread among the people at the time of the spread of the disease, for example due to road closures and other restrictions on the lives of ordinary people?

6. How long was the restriction in your district?

7. If you had cattle or other livestock in your household, did the animals have to be sold? Was it difficult to sell them to the slaughterhouse later?

8. Describe the main treatments in addition to movement restrictions? How were diseased cattle identified (measuring temperature, etc) when there were no external signs of disease?

9. If animals had died in collective farm or in household, what happened next?

10. Do you remember other control magic rituals in addition to official arrangements (treatment schemes) to protect animals in a collective farm or household? (a) juniper smoke was produced; (b) metals (mercury, iron, silver, etc.) were placed in front of the table door; c) holy water was used; d) holy trees were used (juniper, rowan); e) icons were used; (f) the blessing of the animals or the table was used; (g) the animals were given ritual drinks (eg in which silver was inserted); h) other (please specify)

11. Did additional information such as instructions, posters, etc. sent to the farms after the outbreak was over?

12. How many years after the outbreak did the so-called inspections in farms took place?

13. Were there any cases where the disease was transmitted to humans (as H. Mikk highlights it in local newspapers in 1956)?

14. Do you remember any situations where movement restrictions were disregarded? What was the penalty?

Details of the interviewee:

Date of birth

Sex

Acquired specialty

Occupation during the FMD outbreak

District (state)
